# Novel Phenotypes and Deep Intronic Variant Expand TH‐Associated Dopa‐Responsive Dystonia Spectrum

**DOI:** 10.1002/acn3.70013

**Published:** 2025-03-27

**Authors:** Xiaosheng Zheng, Chenxin Ying, Nan Jin, Jinghong Ma, Xinhua Wan, Xunhua Li, Wei Luo

**Affiliations:** ^1^ Department of Neurology The Second Affiliated Hospital, Zhejiang University School of Medicine Hangzhou Zhejiang China; ^2^ Department of Neurology Xuanwu Hospital of Capital Medical University Beijing China; ^3^ Department of Neurology Peking Union Medical College Hospital Beijing China; ^4^ Neurology Department The First Affiliated Hospital, Sun‐Yet Sen University Guangzhou China

**Keywords:** deep intronic variant, dopa‐responsive dystonia, *TH*

## Abstract

Approximately 20% of dopa‐responsive dystonia (DRD) cases remain genetically unresolved. Using whole‐genome sequencing, we identified two *TH* variants in a young DRD patient, including a novel deep intronic variant. Minigene assays confirmed that this variant causes aberrant splicing. The patient exhibited an atypical disease progression compared with typical TH‐associated DRD cases, presenting with generalized dystonia, episodic hypotonia, Parkinsonism, and oromandibular dyskinesias. These findings, including the first known documented deep intronic *TH* variant, expand our understanding of TH‐associated DRD's phenotypic and genotypic spectrum, aiding clinical evaluation.

## 1 Introduction

Dopa‐responsive dystonia (DRD) encompasses a spectrum of rare, genetically and clinically diverse monogenic dystonias caused by variants in *GCH1*, *TH*, *SPR*, *PTS*, and *QDPR* genes. According to the DRD MDSGene review, clinical characteristics such as an early age of onset, robust response to levodopa, the presence of dystonia, and diurnal symptom fluctuations are indicative of DRD [1]. However, challenges in diagnosis arise with non‐GCH1 DRD patients who initially present symptoms like hypotonia, mimicking cerebral palsy, and other atypical presentations characterized by low rates of diurnal fluctuations and oculogyric crises. Indeed, diagnostic delays averaging 5 years were common for DRD patients [1]. Nearly all patients with delayed diagnosis are ultimately identified using molecular genetic methods [2, 3], yet up to 20% of DRD patients test negative for pathogenic variants in the primary genes [4].

In our investigation, we detailed the disease progression of a TH‐associated DRD patient, accompanied by two newly documented phenotypes: episodic hypotonia and oromandibular dyskinesias. Furthermore, we identified and functionally characterized the first deep intronic variant in the *TH* gene, confirming its pathogenicity. These findings expand both our phenotypic and genotypic understanding of TH‐associated DRD, aiding future clinical evaluations of DRD patients.

## 2 Materials and Methods

### 2.1 Subjects and Genetic Study

**FIGURE 1:**
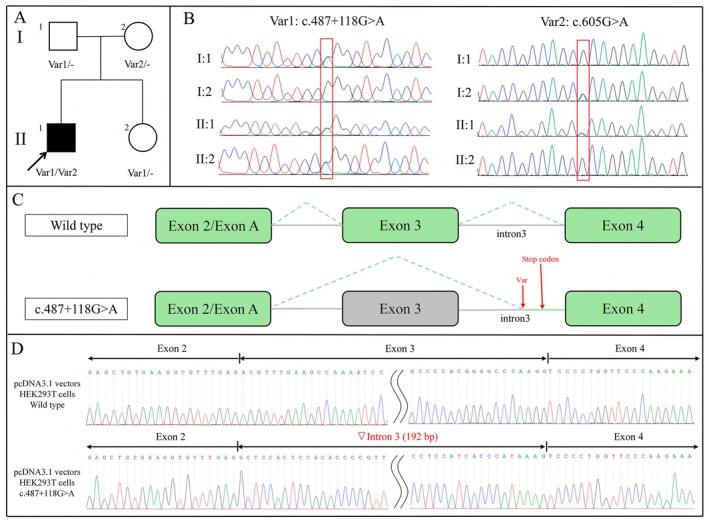
Family pedigree and genetic analyses. (A) Pedigree of the family: The proband (II:1) was identified as a compound heterozygous carrier of *TH* variants—Var1: c.487+118G>A and Var2: c.605G>A. Affected and unaffected family members are distinguished accordingly. (B) Sanger sequencing: Displays the sequencing chromatograms for *TH* variants. The left panel depicts Var1 (c.487+118G>A) observed in individuals I:1, II:1, and II:2, while the right panel shows Var2 (c.605G>A) found in individuals I:2 and II:1. (C) Splice site structure: Minigene assay demonstrates that the c.487+118G>A variant results in exon 3 skipping and intron 3 retention, generating a premature termination codon and a truncated protein. (D) Sanger Sequencing Results of RT‐PCR Products for the pcDNA3.1 Minigene Vector in HEK293T cells: The wild‐type transcript shows normal splicing across Exon 2 (222 bp), Exon 3 (175 bp), and Exon 4 (89 bp). The mutant transcript demonstrates aberrant splicing with exon 3 omission and a 192 bp inclusion from intron 3, resulting in a spliced product of Exon 2 (222 bp), ▽intron3 (192 bp), and Exon 4 (89 bp).

Participants from the family were recruited from the Department of Neurology at Zhejiang University's Second Affiliated Hospital, and were examined face to face by at least two movement disorder specialists using established criteria [1]. The study received approval from the Ethics Committee of the Zhejiang University's Second Affiliated Hospital. The proband (II:1 in Figure 1A) underwent whole‐genome sequencing (WGS) at our institution, which was processed via our in‐house pipeline (see Supporting Information Methods). All variants of genes related to dystonia and Parkinsonism were filtered with allele frequencies ≤ 0.01. SpliceAI was utilized to predict aberrant splicing in the intronic regions of genes [5]. Manta was applied to detect structural variations in the WGS data.

### 2.2 Minigene‐Based Splice Assay

The impact of the deep intronic *TH* variant (c.487+118G>A) was evaluated in vitro using minigene‐based splicing assays. Constructs of both wild‐type and mutant alleles were employed, as previously described [6]. Wild‐type and mutant minigenes were constructed using pcMINI‐C and pcDNA3.1 vectors. The minigene vectors for the deep intronic variant included either the complete intron or a segment of the intron adjacent to its exons (Figure S1). Mutant vectors specific for c.487+118G>A were constructed using DNA from patients. Details of nucleotide primers are provided in Table S1. Both wild‐type and mutant vectors were independently transfected into HeLa and HEK293T cells. After 48 h, transfected cells were harvested using trypsin. Total RNA was then extracted and analyzed by RT‐PCR and gel electrophoresis. Each band of interest was subsequently sequenced using Sanger sequencing.

## 3 Results

### 3.1 Clinical Finding of the Patient

The proband (II:1) was a 16‐year‐old Chinese adolescent with generalized dystonia, episodic hypotonia of the neck, and very mild Parkinsonism. His early developmental milestones were normal. At the age of 2–3 years, he began exhibiting unstable standing. Between the ages of 3 and 4, he developed right leg dystonia, resulting in an abnormal gait. By the age of 6, he experienced episodic hypotonia of the neck presenting like backward arching of the head when fatigued after intense play, requiring rest to recover. These symptoms demonstrated diurnal fluctuations. At the age of 8, dystonia had extended to all four limbs, causing walking difficulties and writing cramps. Subsequently, he was admitted to a movement disorder clinic and diagnosed with episodic dystonia. Treatment with levodopa (50 mg) led to a dramatic improvement; his episodic hypotonia disappeared, and his walking and writing abilities improved, confirming a diagnosis of DRD. Genetic testing at the age of 10 using a dystonia gene panel (Supporting Information Methods) from another institute revealed a heterozygous variant in the *TH* gene (c.605G>A, p.Arg202His). This variant has been reported several times in DRD patients and is classified as likely pathogenic according to the ACMG guidelines (PS3‐strong+PM2‐supporting+PP1‐supporting+PP3‐supporting +PP4‐supporting) [7].

Upon his first visit to our clinic at the age of 16, while on levodopa, he displayed mild right‐sided body dystonia and very subtle bradykinesia in all four limbs (Video S1, Segments 1–2). He also presented with oromandibular dyskinesias, affecting the lower face, mouth, and jaw (Video S1, Segments 1–2). No abnormal eye movements or spasticity were observed. He reported no sleep disorders, cognitive decline, or autonomic symptoms. Dystonia would worsen and neck hypotonia would appear if he felt tired or stayed up very late. He could not recall the exact timing of the onset of oromandibular dyskinesias but reported that they did not improve greatly with levodopa. To differentiate drug‐related dyskinesias and confirm the uncommon neck hypotonia, we assessed the patient before and after the administration of medication during the most recent follow‐up visit (Video S1, Segments 3–5). Oromandibular dyskinesias were present both before and after medication with no major changes, confirming the presence of baseline dyskinesias in the patient. Moreover, before medication, the patient was unable to keep his head still, presenting with backward arching of his neck. A physical examination revealed decreased muscle tone in the neck (Video S1, Segment 4), which completely resolved after levodopa administration.

### 3.2 Detection and Functional Analysis of Deep Intronic Variant in *TH* Gene

Considering that the previous dystonia panel did not include structural variations of *GCH1* and *TH* and did not exclude variants related to Parkinsonism, we performed WGS to conduct a more detailed investigation. None of the variants were detected except for two variants in the *TH* gene. One was the aforementioned missense variant (c.605G>A, p.Arg202His), and the other was a novel deep intronic variant (c.487+118G>A). The deep intronic variant was rare in the population (0.0000157 from gnomAD) and was predicted to affect the splice site by SpliceAI (Δscore = 0.91). Familial segregation analysis showed that the c.605G>A variant (p.Arg202His) was inherited from his mother (I:2 in Figure 1A) and the c.487+118G>A variant from his father (I:1 in Figure 1A), while his healthy 7‐year‐old sister (II:2 in Figure 1A) only carried a heterozygous variant of c.487+118G>A (Figure 1B). This result confirmed that the proband was a compound heterozygous carrier of these two *TH* variants.

Expression level of *TH* gene was observed to be extremely low in whole blood. Therefore, a minigene splice assay was conducted to assess the impact of the c.487+118G>A variant on transcript splicing. RT‐PCR products from both wild‐type and mutant vectors in HeLa and HEK293T cells were examined on gel electrophoresis, indicating bands from mutant vectors were subtly longer than those of wild‐type vectors (Figure S2). Sanger sequencing of RT‐PCR products from mutant vectors demonstrated an aberrant splice isoform of *TH* with exon 3 skipping and partial retention of intron 3 (Figures 1C,D and S3). The retained intron generated a premature termination codon, resulting in a truncated protein of 141 amino acids (Supporting Information Results). Consequently, the c.487+118G>A variant was classified as pathogenic according to ACMG guidelines (PVS1‐very strong+PM2‐supporting+PP3‐supporting+PP4‐supporting). The patient was finally genetically diagnosed with DRD, caused by pathogenic compound heterozygous *TH* variants, and continued to respond well to levodopa treatment.

## 4 Discussion

DRD is a complex disease with a wide range of phenotypic variability, making it difficult to recognize clinically and often leading to missed diagnoses. Specific symptoms may delineate DRD subtypes; for instance, autonomic disturbances are common in TH‐ and PTS‐associated DRD, whereas sleep disturbances and oculogyric crises are more characteristic of SPR‐associated DRD [1]. Typically, TH‐associated DRD patients first develop hypotonia, followed by dystonia, and approximately 36% of these patients later exhibit Parkinsonism [1]. However, our patient's progression deviated significantly from this pattern, as he initially presented with dystonia, followed by episodic hypotonia, and subsequently oromandibular dyskinesias and Parkinsonism, without manifestation of the specific signs observed. This finding highlights the potential complexity and variability of initial presentations and disease progression in TH‐associated DRD. This complexity may be attributed to the presence of the novel deep intronic variant, though further data collection and analysis are required to confirm this.

To our knowledge, this study is the first to report two new phenomena in TH‐associated DRD: episodic hypotonia of the neck and oromandibular dyskinesias. Typically, DRD‐associated hypotonia presents as axial hypotonia responsive to levodopa treatment [8]. In our case, episodic hypotonia predominantly occurred in the neck, typically following strenuous activity or sleep deprivation, and responded well to levodopa. This is consistent with the typical response of axial hypotonia in DRD patients and confirms it as a symptom of TH‐associated DRD. Previously, mild dyskinesias were frequently observed in DRD patients on higher doses of levodopa compared with non‐dyskinetic DRD patients [9]. These dyskinesias resolved upon the reduction or discontinuation of medication [10]. However, the oromandibular dyskinesias observed in our patient persisted both before and after levodopa treatment, which has not previously been reported in DRD patients. This baseline dyskinesia may be linked to the underlying *TH* deficiency, but further case reports and mechanistic studies are needed to clarify this relationship. Therefore, future assessments of episodic hypotonia and oromandibular dyskinesias in TH‐associated DRD patients may be warranted, and these symptoms might even be considered potential indicators of TH‐associated DRD.

Most reported splice site *TH* variants were located at canonical splice sites, with only two reports of non‐canonical splice site variants (c.1201‐24T>A and c.645‐34C>A) identified through Sanger sequencing [11, 12]. Due to the low expression of the *TH* gene in blood, only one study confirmed aberrant splicing using RNA isolated from cultured fibroblasts for c.1201‐24T>A. Advances in sequencing technology have highlighted deep intronic variants as potential key factors in uncovering the “missing heritability” of neurological diseases [13]. For DRD‐associated genes, a single study reported a homozygous deep intronic variant (c.541+347A>G) in the *GCH1* gene [14]. This variant, not listed in the gnomAD dataset, was predicted to have minimal impact on splicing events, requiring further functional analysis to establish its pathogenicity. In the present case, we used a minigene assay to confirm that this deep intronic *TH* variant results in a truncated protein of 141 amino acids, aligning with the characteristics of *TH* gene loss of function [15], thus broadening the genetic landscape of TH‐associated DRD and enhancing its clinical interpretation.

## Author Contributions

**Xiaosheng Zheng:** study design, execution, statistical analysis, writing of the first draft, editing of the manuscript. **Chenxin Ying:** study design, execution, writing of the first draft, editing of the manuscript. **Nan Jin:** study execution, editing of the manuscript. **Jinghong Ma:** study execution, statistical analysis, editing of the manuscript. **Xinhua Wan:** study execution, editing of the manuscript. **Xunhua Li:** study design, editing of the manuscript. **Wei Luo:** study design, execution, statistical analysis, writing of the first draft, editing of the manuscript.

## Conflicts of Interest

The authors declare no conflicts of interest.

## Supporting information


**Figure S1.** Schematic of the pcMINI‐C and pcDNA3.1 minigene vectors.
**FIGURE S2**. Agarose gel electrophoresis of RT‐PCR products.
**FIGURE S3**. Analysis of the remaining RT‐PCR products through Sanger sequencing from minigene vectors.
**TABLE S1**. Nucleotide primers for mutagenesis, amplification of mutant vectors, and restriction endonuclease.

**Video S1.** Segments 1 and 2 were recorded during the patient’s first visit to our clinic while on levodopa treatment. Segments 3, 4, and 5 were recorded during the most recent follow‐up visit, with Segments 3 and 4 captured before levodopa administration and Segment 5 after taking levodopa. Segment 1: This segment displays the proband performing finger tapping and toe tapping exercises, illustrating bradykinesia in all four limbs. Additionally, there is subtle right‐sided body dystonia and oromandibular dyskinesias evident during the movements. The patient shows subtle dyskinesias of the fingers when performing tasks with the opposite hand. Segment 2: This segment shows the patient walking, highlighting an extremely subtle dystonic gait in the lower limbs, indicative of mild muscle tone disturbances. Segment 3: Before levodopa administration, the proband performs finger tapping and toe tapping exercises, revealing obvious bradykinesia in all four limbs, neck retroflexion, and oromandibular dyskinesias. Segment 4: Prior to levodopa treatment, decreased neck muscle tone is evident, with the patient unable to maintain an upright position, leading to him falling backward or forward. Segment 5: After levodopa administration, the patient’s limb movements show significant improvement compared to the pre‐treatment state, and he can maintain a normal neck posture.

## Data Availability

The data that support the findings of this study are available from the corresponding author upon reasonable request.
