# Supplementary material for: Novel Phenotypes and Deep Intronic Variant Expand TH‐Associated Dopa‐Responsive Dystonia Spectrum
Source: Ann Clin Transl Neurol. 2025 Mar 27;12(5):1093–6. doi: 10.1002/acn3.70013 (PMC12093331; doi:10.1002/acn3.70013)
Supplement: Supplementary file 1 — Figure S1. Schematic of the pcMINI‐C and pcDNA3.1 minigene vectors. FIGURE S2. Agarose gel electrophoresis of RT‐PCR products. FIGURE S3. Analysis of the remaining RT‐PCR products through Sanger sequencing from minigene vectors. TABLE S1. Nucleotide primers for mutagenesis, amplification of mutant vectors, and restriction endonuclease. [file ACN3-12-1093-s001.docx]

**Supplementary Appendix**

This supplementary appendix is offered by the authors to furnish readers with further insights and details pertaining to their research.

**TABLE OF CONTENTS**

**SUPPLEMENTARY METHODS …………………………………………………………………1-2**

**SUPPLEMENTARY FIGURES ……………………………………………………………………3-4**

**SUPPLEMENTARY TABLES ……………………………………………………………………5-5**

**SUPPLEMENTARY RESULTS ……………………………………………………………………6-6**

**SUPPLEMENTARY REFERENCES ………………………………………………………………7-7**

**Supplementary Methods**

**Dystonia gene panel**

The dystonia gene panel was designed based on prior literature^1^ and technical support from Illumina. The specific genes included are no longer accessible. The targeted regions for probe hybridization capture encompass the exons of these genes and the adjacent intronic regions within a 50 bp range.

**Detailed WGS analysis pipeline**

A custom, in-house bioinformatics pipeline was employed to analyze the sequencing data extracted from WGS and WES. The software implemented was set to default parameters, with exceptions made only for specialized annotations.

The steps included:

1. Quality control of FASTQ files was carried out using fastp version 0.23.2.

2. Paired-end reads were mapped to the UCSC hg38 reference using BWA-mem2 version 2.2.1, generating a Sequence Alignment/Map (SAM) file. The reference was obtained from (http://hgdownload.soe.ucsc.edu/goldenPath/hg38/bigZips/).

3. The SAM file was converted to a Binary Alignment Map (BAM) file with the help of Samtools version 1.16, using both the sorting and indexing modules.

Steps 4 to 7 were performed in accordance with the guidelines of the Genome Analysis Toolkit (GATK) best practice pipeline:

4. Duplicates in the BAM file were marked using the GATK version 4.3.0.0 MarkDuplicates module.

5. Base Quality Score Recalibration (BQSR) was applied to the BAM file with the GATK version 4.3.0.0 BQSR module.

6. Variants were called per sample using the GATK version 4.3.0.0 HaplotypeCaller module, creating a raw Variant Call Format (VCF) file. For WES files, the '-L bedfile' option was utilized.

7. Variant normalization and splitting was carried out using VT normalize and decompose.

8. Variants were annotated against genes using ANNOVAR with the ClinVar, ExAC_EAS, and gnomAD datasets.

9. Structural variants were identified with the aid of CNVkit version 0.9.10, using the '-L bedfile' option for WES files.

10. Known related genes were filtered with the script.

**Supplementary Figure 1.** Schematic representation of pcMINI-C and pcDNA3.1 minigene vectors.


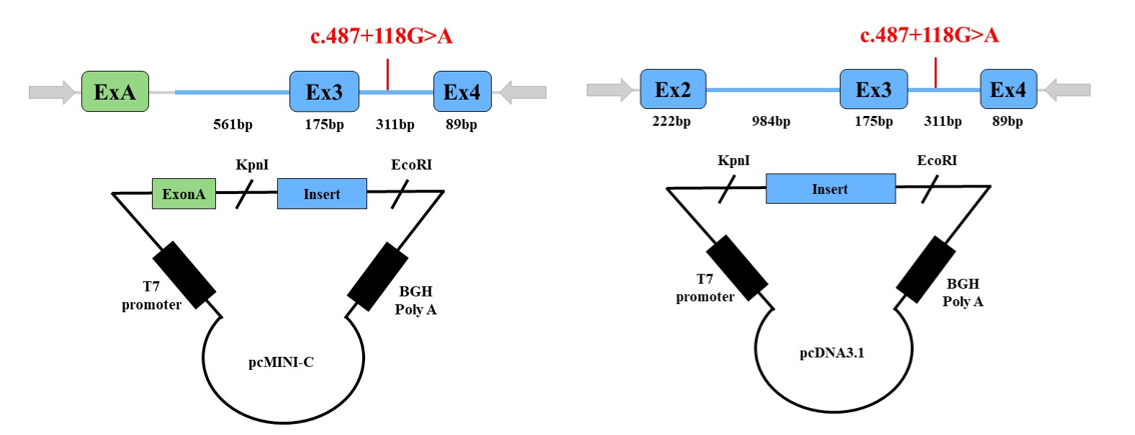


**Supplementary Figure 2.** Agarose Gel Electrophoresis of RT-PCR Products. This panel illustrates the RT-PCR products derived from both wild-type and mutant alleles of c.487+118G>A, separated by agarose gel electrophoresis. Subtle differences between the wild-type and mutant product bands are discernible, attributed to the similar lengths of exon 3 skipping (175 bp) and partial retention of intron 3 (192 bp).
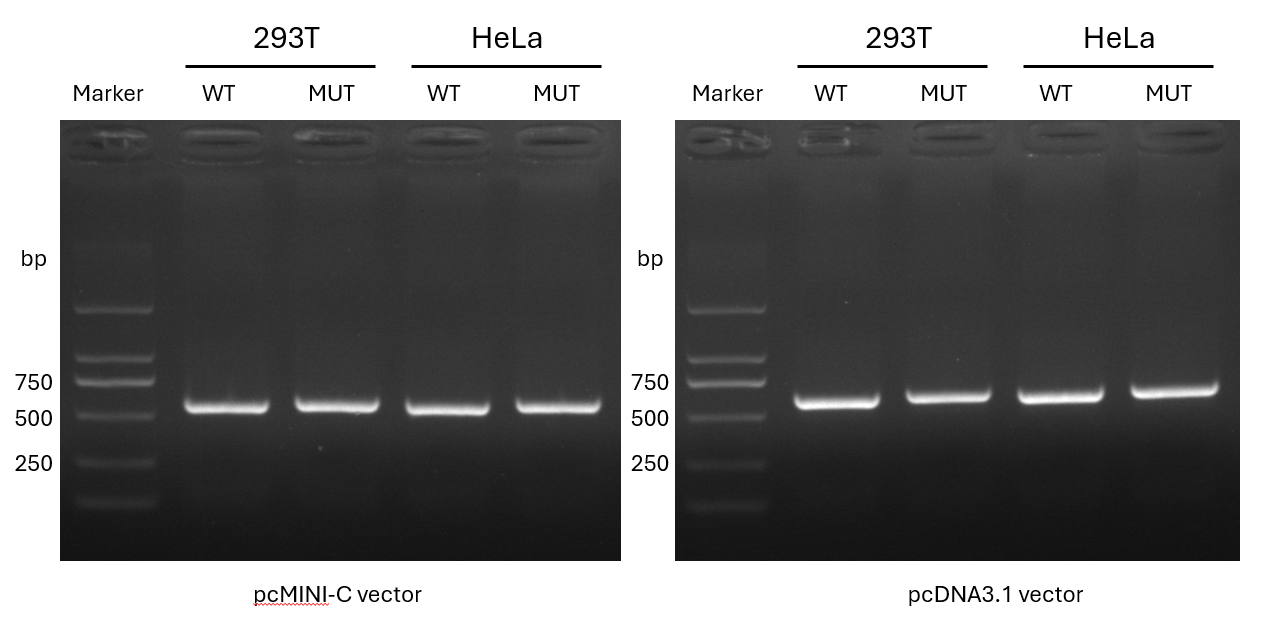


**Supplementary Figure 3.** Analysis of RT-PCR Products via Sanger Sequencing from Minigene Vectors. This figure shows the sequencing results for both wild-type and mutant minigene vectors expressed in various cell lines. All wild-type transcript shows normal splicing across Exon 2 (222 bp), Exon 3 (175 bp), and Exon 4 (89 bp). All mutant transcript demonstrates aberrant splicing with exon 3 omission and a 192 bp inclusion from intron 3, resulting in a spliced product of Exon 2 (222 bp), ▽intron3 (192 bp), and Exon 4 (89 bp).


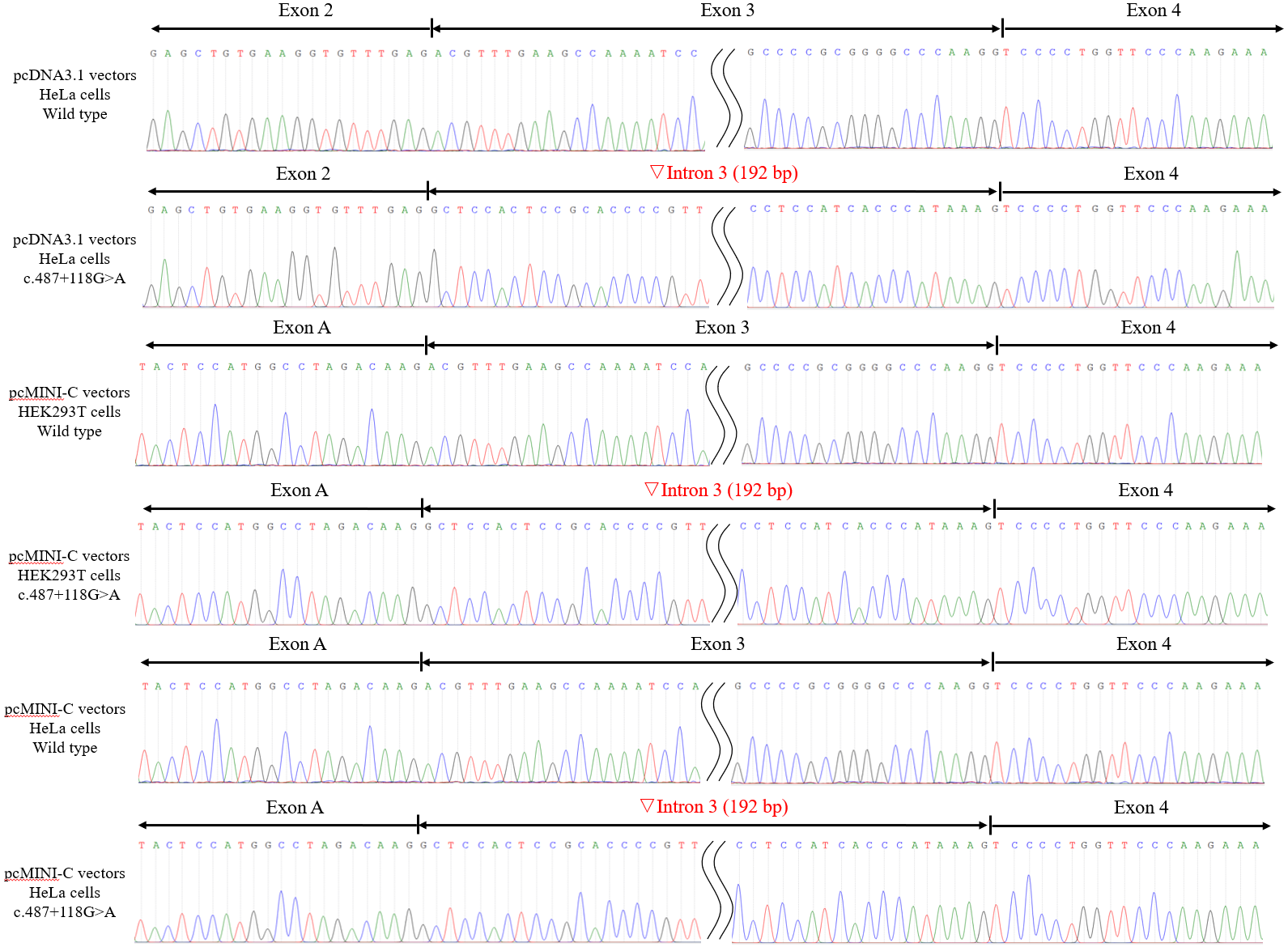


**Supplementary Table 1**. Nucleotide Primers for Mutagenesis, Amplification of Mutant Vectors, and Restriction Endonuclease Details

| Primer Name | Primer Sequence |
| --- | --- |
| 1435-TH-F | 5’- gtgagaaagggtcccctgtg-3’ |
| 1621-TH-F | 5’- tctctgtctgtctgctcgcc-3’ |
| 3928-TH-R | 5’- ccctcacTGCCTGTACTGGA-3’ |
| 4186-TH-R | 5’- gttggaggatggacaggagg-3’ |
| pcMINI-C-TH-KpnI-F | 5’- ggtaGGTACCacccagctcccggaagaagc-3’ |
| pcMINI-C-TH-EcoRI-R | 5’- TGCAGAATTCCGGGTGGTCCAAGTCCAGGT-3’ |
| pcDNA3.1-TH-KpnI-F | 5’- GCTTGGTACCatgTCCCCGCGGTTCATTGGGCG-3’ |
| pcDNA3.1-TH-EcoRI-R | 5’- TGCAGAATTCCGGGTGGTCCAAGTCCAGGT-3’ |
| pcMINI-C-F | 5’- CTAGAGAACCCACTGCTTAC-3’ |
| pcMINI-C-R | 5’- TGCAGAATTCCGGGTGGTCCAAGTCCAGGT-3’ |
| pcDNA3.1-F | 5’- CTAGAGAACCCACTGCTTAC-3’ |
| pcDNA3.1-R | 5’- TGCAGAATTCCGGGTGGTCCAAGTCCAGGT-3’ |

**Supplementary Result**

**Detailed Sequencing of Mutant Transcript.** This panel displays the sequencing results for the mutant transcript, with Exon 2, Exon 4, and retained Intron 3 each highlighted in distinct colors for clarity. A premature termination codon within the retained Intron 3 is marked in red. The presence of this codon leads to the formation of a truncated protein comprising 141 amino acids, illustrating the impact of aberrant splicing on protein structure.

ATCG presents exon2

ATCG presents exon4

ATCG presents retained intron 3

TAG presents premature termination codon

TCCCCGCGGTTCATTGGGCGCAGGCAGAGCCTCATCGAGGACGCCCGCAAGGAGCGGGAGGCGGCGGTGGCAGCAGCGGCCGCTGCAGTCCCCTCGGAGCCCGGGGACCCCCTGGAGGCTGTGGCCTTTGAGGAGAAGGAGGGGAAGGCCGTGCTAAACCTGCTCTTCTCCCCGAGGGCCACCAAGCCCTCGGCGCTGTCCCGAGCTGTGAAGGTGTTTGAGGCTCCACTCCGCACCCCGTTTTGCTACACATCCGTGTCCGGGCCTGGGGCCACTCCAGGATCCCCCCGCAGCTCTCACAGCCCCGGCTGCCTCTGCCCCCCGGAAGTCTTGTAGGGGAGGCTGCTTCAAGGTGGGTGACACAGCCCCACGGCTCCGAGCTCACCAAGATCTCTTCCTCCATCACCCATAAAGTCCCCTGGTTCCCAAGAAAAGTGTCAGAGCTGGACAAGTGTCATCACCTGGTCACCAAGTTCGACCCTGACCTGGACTTGGACCACCCG

**Supplementary References**

1. Mamanova L, Coffey AJ, Scott CE, et al. Target-enrichment strategies for next-generation sequencing. Nat Methods. 2010;7(2):111-118.
